# ﻿*Dimorphostylispilocorpus* sp. nov. (Crustacea, Cumacea, Diastylidae), a new cumacean from Korean waters

**DOI:** 10.3897/zookeys.1193.115782

**Published:** 2024-02-27

**Authors:** Sung-Hyun Kim, Taekjun Lee

**Affiliations:** 1 Department of Biological Sciences, Dankook University, Cheonan, Republic of Korea; 2 Department of Animal Resources Science, Sahmyook University, Seoul, Republic of Korea; 3 Marine Biological Resource Institute, Sahmyook University, Seoul, Republic of Korea

**Keywords:** Cumacea, Diastylidae, *Dimorphostylis*, identification key, morphology, new species, taxonomy

## Abstract

A new species of Cumacea belonging to the genus *Dimorphostylis* Zimmer was collected from the Dokdo and Ulleung Islands in the East Sea of Korea. The new species, *Dimorphostylispilocorpus***sp. nov.**, can be distinguished from all other *Dimorphostylis* species by the combination of the body surface covered with numerous slender simple setae; carapace with one transverse, a pair of frontal, and three pairs of oblique ridges; three pairs of oblique ridges connected on a submedian carina; telson of the female with 1 pair of short simple and 1 short, stout simple seta centrally; 1 pair of stout simple and 3 pairs of short simple setae on the post-anal section; pleonite 5 of male with 1 spiniform seta on the ventral margin; post-anal section with 2 pairs of stout simple setae bearing a single subterminal setule on both sides; terminal margin with 3 stout simple setae; and a central seta slightly longer than the outer pair of setae. Full illustrations of the new species, including the mouthparts, are given in this paper. A key to the Korean species of *Dimorphostylis* is also provided.

## ﻿Introduction

The family Diastylidae Bate, 1856 is currently composed of 25 genera and approximately 355 species worldwide (WoRMS 2023). Among the 25 genera, *Dimorphostylis* Zimmer, 1921 is the fourth most speciose genus, following *Diastylis* Say, 1818, *Makrokylindrus* Stebbing, 1912 and *Leptostylis* G.O. Sars, 1869. The genus *Dimorphostylis* was established by Zimmer (1921) for *Dimorphostylisasiatica* from Japanese waters, and 33 species have been reported. *Dimorphostylis* is distributed in the Indo-West Pacific and the coast of Australia (Akiyama 2011; Gerken 2014). All known *Dimorphostylis* are shallow water inhabitants, with the deepest previous record of 918 m in *Dimorphostylisbrevicarpus* Akiyama, 2011. Up to now, nine *Dimorphostylis* species have been recorded from Korean waters: *Dimorphostylisacroplicata* Harada, 1960, *D.asiatica* Zimmer, 1921, *D.breviplicata* Lee & Lee, 2012, *D.echinata* Gamô 1962, *D.hirsuta* Gamô 1960, *D.longicauda* Gamô 1962, *D.manazuruensis* Gamô 1960, *D.namhaedoensis* Lee & Lee, 2000 and *D.valida* Harada, 1960 (Kang and Lee 1995; Hong et al. 1998; Lee and Lee 2002, 2007, 2012; Lee et al. 2003). In this study, we describe and illustrate a new species of *Dimorphostylis* from Korean waters. A key to the Korean species of the genus *Dimorphostylis* is also provided.

## ﻿Material and methods

The specimens were collected using a light trap (Holmes and O’Connor 1988; Kim 1992) from the Dokdo and Ulleung Islands (East Sea), Korea. The collected specimens were fixed in 80% ethanol, moved to the laboratory, and stored in 95% ethanol. The specimens were identified with a stereomicroscope (Model SZX12; Olympus, Japan). Photographs of the whole body were taken with a microscope equipped with a digital camera (eXcope T500; DIXI Science, Korea) and complemented by Helicon Focus v. 7.7.5 (Helicon Soft Ltd., Kharkiv, Ukraine). The body length was measured from the anterior tip of the carapace to the posterior end of pleonite 6. The lengths of the appendages were measured along the mid-line of each appendage. The whole body was drawn using a stereomicroscope (Olympus SZX12) with a drawing tube. Later, the samples were transferred to glycerin for dissection under a stereomicroscope (Olympus SZX12). Drawing of the appendages was performed using a light microscope (Model BX51; Olympus, Japan) with a drawing tube. Type specimens were deposited at the National Institute of Biological Resources (NIBR), Incheon, Korea.

## ﻿Taxonomy

### ﻿Genus Dimorphostylis

Taxon classificationAnimaliaCumaceaDiastylidae

Zimmer, 1921

08E7BFAB-C37F-5001-9632-3BF86AD6D2D7

#### Type species.

*Dimorphostylisasiatica* Zimmer, 1921.

#### Diagnosis.

***Female and subadult male*.** Maxilliped 3 with exopod. Pereopods 3–4 without exopods in female and with exopods in subadult male. ***Adult male*.** Bases of pereopods 2–4 expanded, with exopod.

#### Species composition.

*Dimorphostylisacroplicata* Harada, 1960, *D.asiatica* Zimmer, 1908, *D.australis* Foxon, 1932, *D.bathyelegans* Akiyama, 2011, *D.brevicarpus* Akiyama, 2011, *D.brevicaudata* (Zimmer, 1903), *D.breviplicata* Lee & Lee, 2012, *D.colefaxi* Hale, 1945, *D.cornigera* Harada, 1960, *D.coronata* Gamô, 1960, *D.cottoni* Hale, 1936, *D.echinata* Gamô, 1962, *D.elegans* Gamô, 1960, *D.gibbosa* Harada, 1960, *D.hirsuta* Gamô, 1960, *D.horai* Kurian, 1956, *D.inauspicata* Hale, 1945, *D.latifrons* Harada, 1960, *D.longicauda* Gamô, 1962, *D.longitelson* Kurian, 1963, *D.maledivensis* Mühlenhardt-Siegel, 1996, *D.manazuruensis* Gamô, 1960, *D.namhaedoensis* Lee & Lee, 2002, *D.nordaustraliana* Gerken, 2014, *D.quadriplicata* Gamô, 1960, *D.roccatagliatai* Gerken, 2014, *D.sculpturensis* Vassilenko & Tzareva, 1990, *D.subaculeata* Hale, 1945, *D.tasmanica* Hale, 1945, *D.tribulis* Hale, 1945, *D.triplicata* Gerken, 2014, *D.valida* Harada, 1960 and *D.vieta* (Hale, 1936).

### ﻿ Dimorphostylis pilocorpus sp. nov.

Taxon classificationAnimaliaCumaceaDiastylidae

B6279EA6-01BE-5CFC-8ED1-F8BA070DB7DC

https://zoobank.org/1A9BEBD2-3F28-4966-8A30-8AFF7EFD89A6

**Figure 1.**
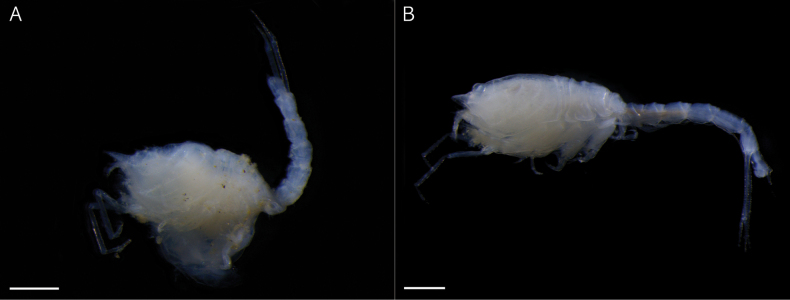
*Dimorphostylispilocorpus* sp. nov. **A** holotype, ovigerous female, 3.51 mm, cat no. NIBRIV0000901416, lateral view **B** paratype, adult male, 4.62 mm, cat no. NIBRIV0000911248, lateral view. Scale bars: 0.5 mm (**A, B**).

**Figure 2.**
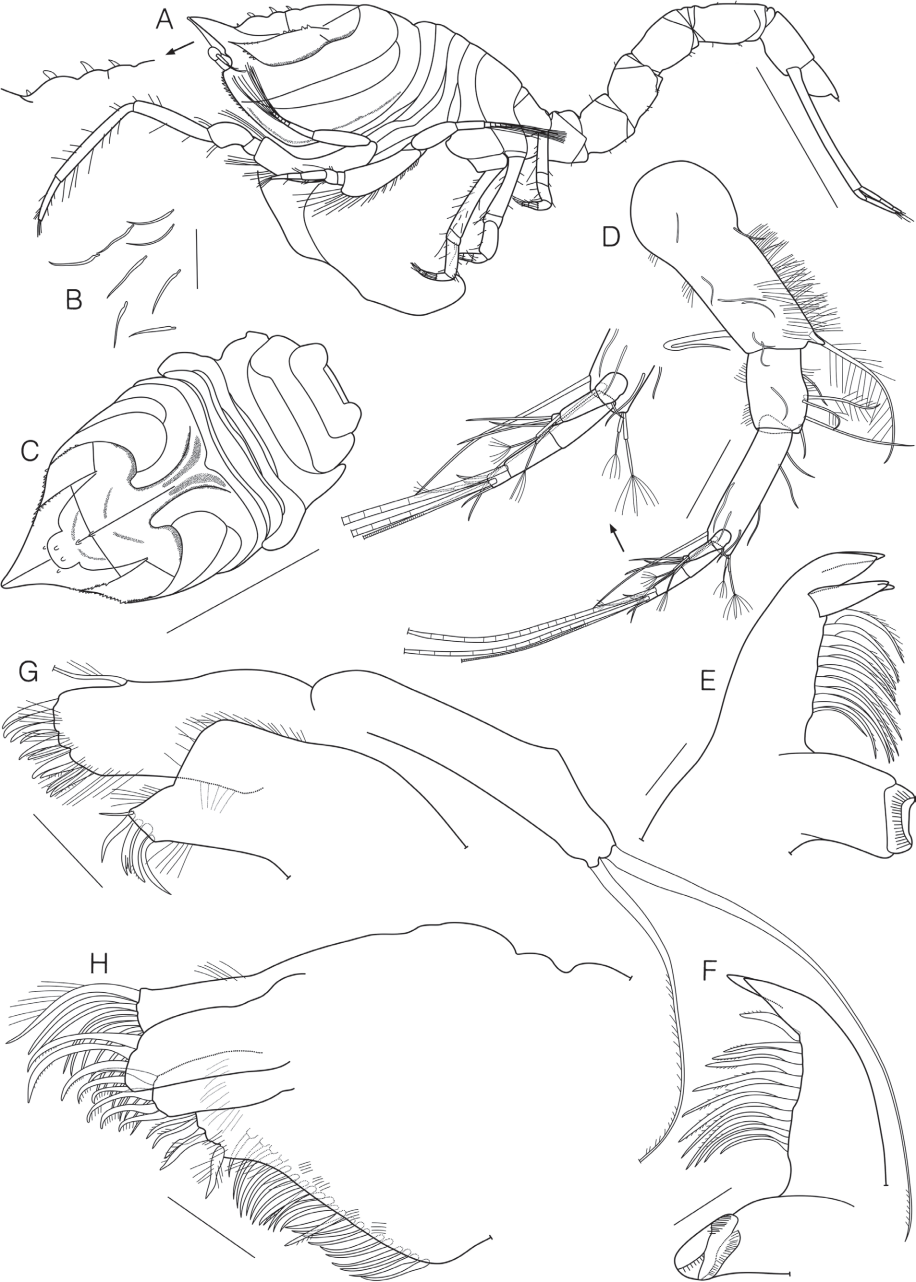
*Dimorphostylispilocorpus* sp. nov., paratype, ovigerous female, 4.42 mm, cat no. NIBRIV0000911249 **A** habitus, lateral view **B** slender simple setae **C** carapace and pereon, dorsal view **D** antenna 1 **E** left mandible **F** right mandible **G** maxilla 1 **H** maxilla 2. Scale bars: 1.0 mm (**A, C**); 0.1 mm (**D**); 0.05 mm (**B, E–H**).

**Figure 3.**
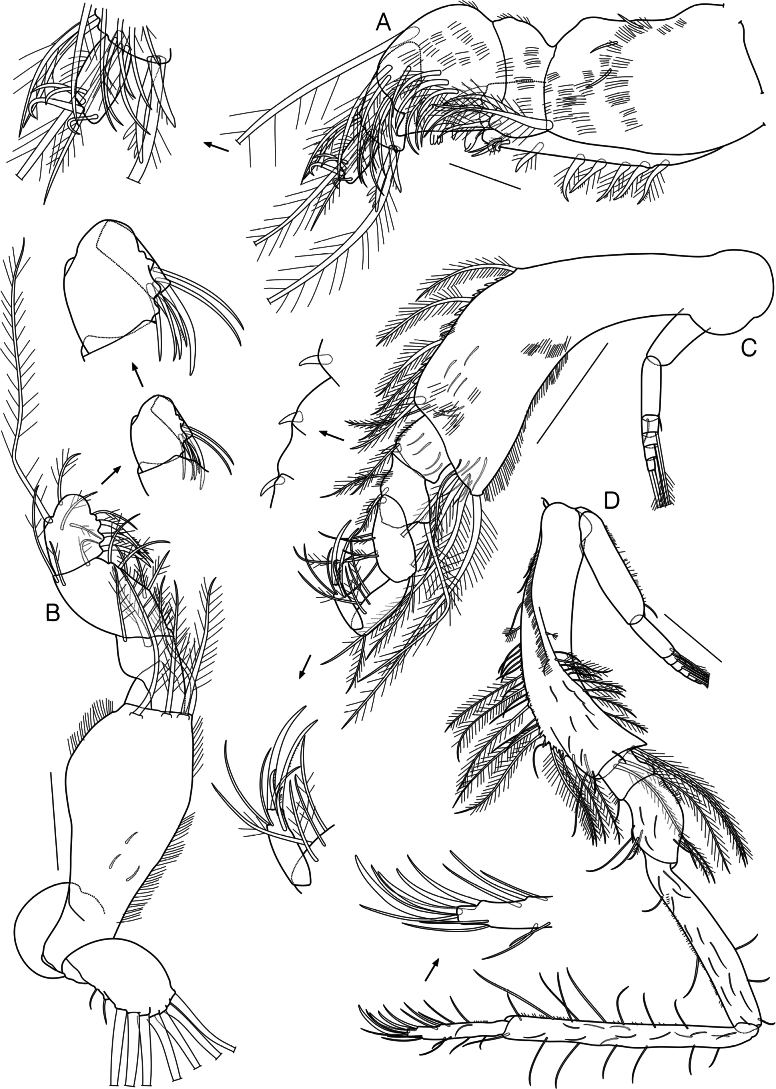
*Dimorphostylispilocorpus* sp. nov., paratype, ovigerous female, 4.42 mm, cat no. NIBRIV0000911249 **A** maxilliped 1 **B** maxilliped 2 **C** maxilliped 3 **D** pereopod 1. Scale bars: 0.2 mm (**C, D**); 0.1 mm (**B**); 0.05 mm (**A**).

**Figure 4.**
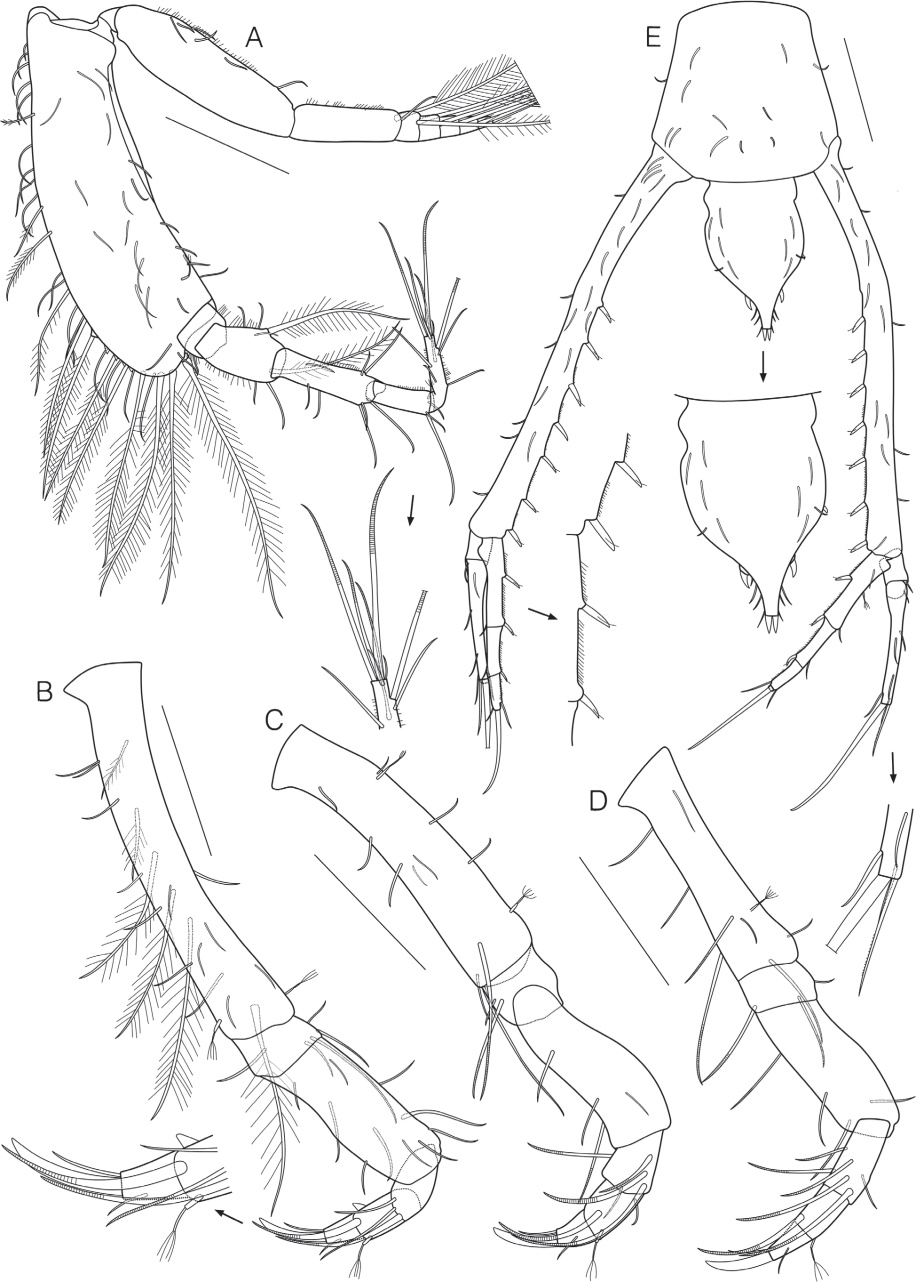
*Dimorphostylispilocorpus* sp. nov., paratype, ovigerous female, 4.42 mm, cat no. NIBRIV0000911249 **A** pereopod 2 **B** pereopod 3 **C** pereopod 4 **D** pereopod 5 **E** telson and uropods. Scale bars: 0.2 mm (**A–E**).

**Figure 5.**
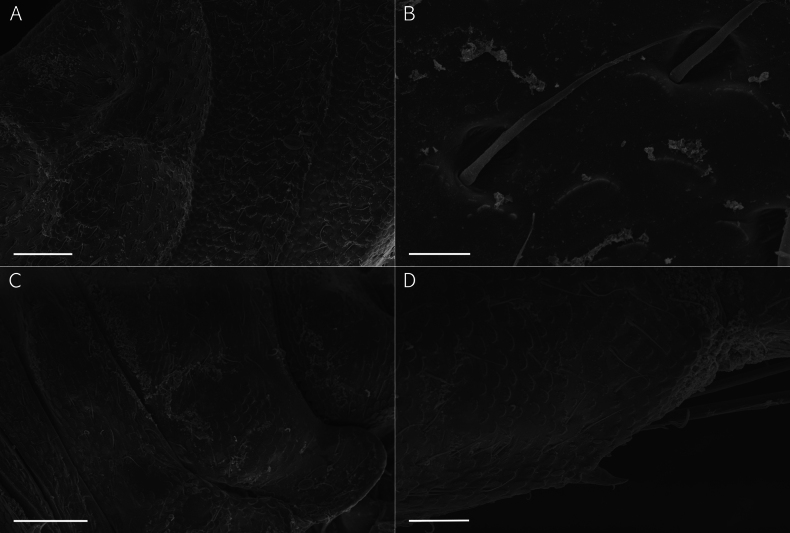
*Dimorphostylispilocorpus* sp. nov., paratype, adult male, 4.46 mm **A** carapace **B** slender simple setae **C** pereon **D** pleonite 5. Scale bars: 100 μm (**A, C**); 50 μm (**D**); 10 μm (**B**).

**Figure 6.**
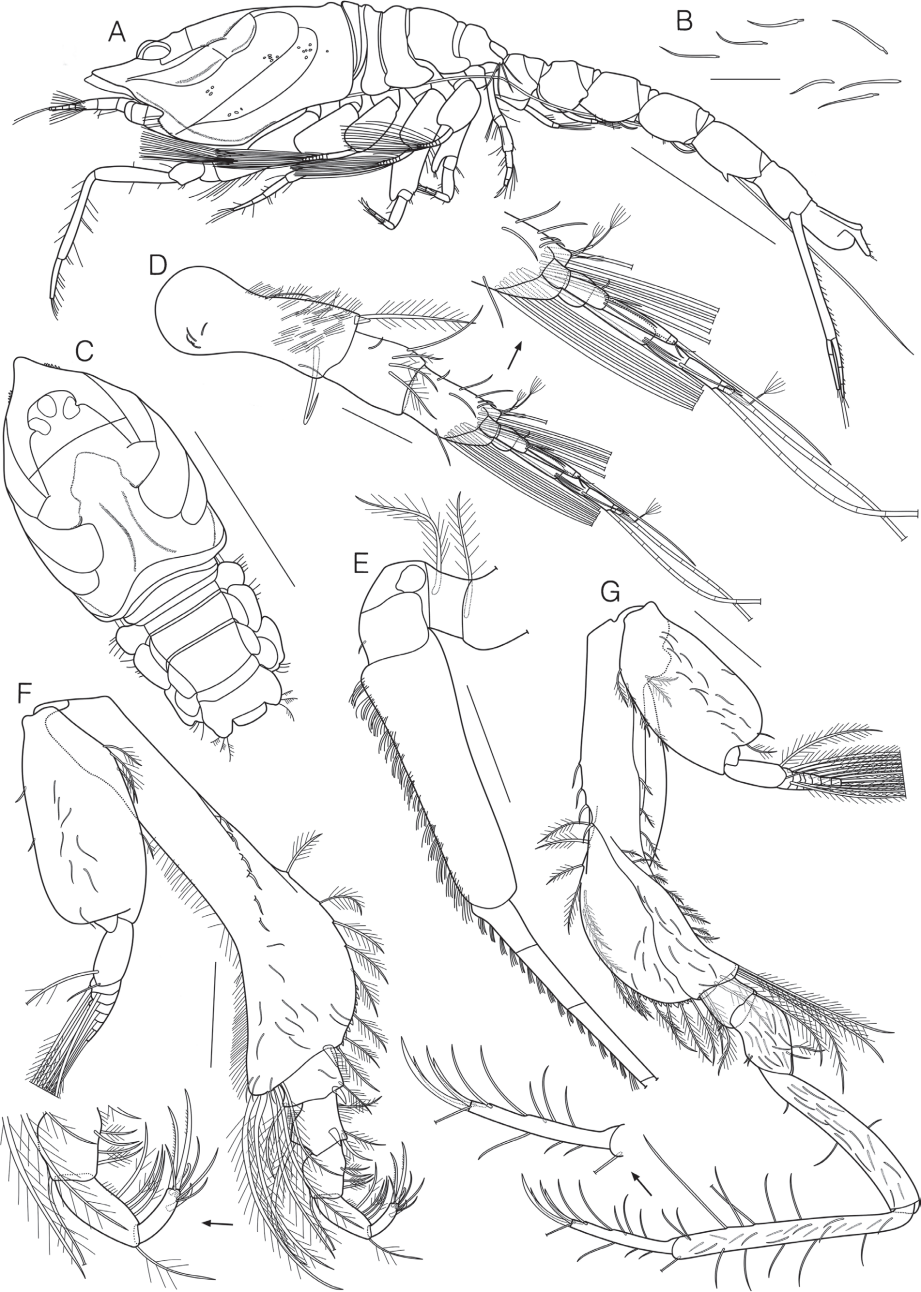
*Dimorphostylispilocorpus* sp. nov., paratype, adult male, 4.45 mm, cat no. NIBRIV0000911250 **A** habitus, lateral view **B** slender simple setae **C** carapace and pereon, dorsal view **D** antenna 1 **E** antenna 2 **F** maxilliped 3 **G** pereopod 1. Scale bars: 1.0 mm (**A, C**); 0.2 mm (**E–G**); 0.1 mm (**D**); 0.05 mm (**B**).

**Figure 7.**
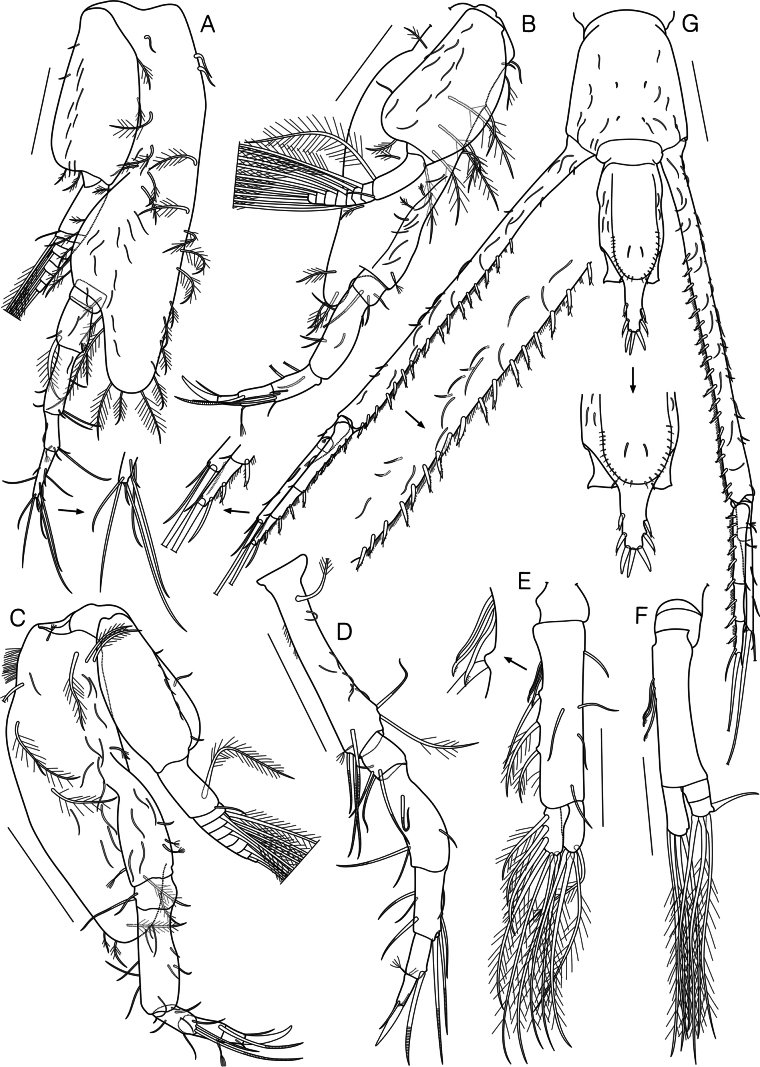
*Dimorphostylispilocorpus* sp. nov., paratype, adult male, 4.45 mm, cat no. NIBRIV0000911250 **A** pereopod 2 **B** pereopod 3 **C** pereopod 4 **D** pereopod 5 **E** pleopod 1 **F** pleopod 2 **G** telson and uropods. Scale bars: 0.2 mm (**A–D, G**); 0.1 mm (**E, F**).

Figs 1 , 2 , 3 , 4 , 5 , 6 , 7 Korean name: Ju-reum-teol-bo-i-hyeong-ol-chaeng-i-sae-u, new

#### Type material.

***Holotype***, ovigerous female, 3.51 mm, cat no. NIBRIV0000901416, Sadong Port, Sadong-ri, Ulleung-eup, Ulleung-gun, Gyeongsangbuk-do, Korea, 37°27'37.1"N, 130°52'35.3"E, 3 July 2022, S.H. Kim collected by light trap. ***Paratypes***, 1 ovigerous female, cat no. NIBRIV0000911249 and 2 adult males, cat no. NIBRIV0000911248, NIBRIV0000911250, data same as holotype (1♂ 1♀ dissected); the remaining paratypes 80♂♂ 12♀♀, cat no. NIBRIV0000911251, data same as holotype.

#### Additional material examined.

1♂, NIBRIV0000901413, Namyang Port, Namyang-ri, Seo-myeon, Ulleung-gun, Gyeongsangbuk-do, Korea, 37°28'00.8"N, 130°49'59.5"E, 28 April 2022, S.H. Kim collected by light trap; 1♂ 1♀, NIBRIV0000901414, Namyang Port, Namyang-ri, Seo-myeon, Ulleung-gun, Gyeongsangbuk-do, Korea, 37°28'00.8"N, 130°49'59.5"E, 3 July 2022, S.H. Kim collected by light trap; 49♂♂, NIBRIV0000901415, Sadong Port, Sadong-ri, Ulleung-eup, Ulleung-gun, Gyeongsangbuk-do, Korea, 37°27'37.1"N, 130°52'35.3"E, 28, April, 2022, S.H. Kim collected by light trap; 9♂♂, NIBRIV0000901417, Dokdo-ri, Ulleung-eup, Ulleung-gun, Gyeongsangbuk-do, Korea, 37°14'38.0"N, 131°51'41.0"E, 30 April 2022, S.H. Kim collected by light trap; 1♂, NIBRIV0000901418, Dokdo-ri, Ulleung-eup, Ulleung-gun, Gyeongsangbuk-do, Korea, 37°14'23.1"N, 131°52'03.5"E, 30 April 2022, S.H. Kim collected by light trap; 5♂♂, NIBRIV0000901419, Dokdo-ri, Ulleung-eup, Ulleung-gun, Gyeongsangbuk-do, Korea, 37°14'17.8"N, 131°52'08.8"E, 6 July 2022, S.H. Kim collected by light trap; 3♂♂ 6♀♀, NIBRIV0000901420, Dokdo-ri, Ulleung-eup, Ulleung-gun, Gyeongsangbuk-do, Korea, 37°14'26.3"N, 131°52'16.6"E, 7 July 2022, S.H. Kim collected by light trap; 2♀♀, NIBRIV0000901421, Cheonbu Port, Cheonbu-ri, Buk-myeon, Ulleung-gun, Gyeongsangbuk-do, Korea, 37°32'27.1"N, 130°52'22.3"E, 4 July 2022, S.H. Kim collected by light trap.

#### Diagnosis.

Body surface covered with numerous slender simple setae, carapace with 1 transverse, 1 pair of frontal, and 3 pairs of oblique ridges. ***Adult female*.** Carapace with 1 pair of small spines respectively on pseudorostral lobe and ocular lobe, 2 small spines on median region of frontal lobe, telson with 1 pair of short simple and 1 short stout simple seta centrally, 1 pair of stout simple and 3 pairs of short simple setae on post-anal section, 2 stout simple setae terminally. ***Adult male*.** Pleonite 5 with 1 spiniform seta on ventral margin, post-anal section with 2 pairs of stout simple setae bearing single subterminal setule on both sides, terminal margin with 3 stout simple setae, central seta slightly longer than outer pair of setae.

#### Description.

**Paratype, ovigerous female, cat no. NIBRIV0000911249.** Body (Fig. 2A) length 4.42 mm. Body surface covered with numerous slender, simple setae (Figs 2B, 5B). Carapace (Fig. 2A, C) 0.3 times as long as body, 1.2 times as long as width, 1.4 times as long as depth, frontal lobe with 1 transverse ridge; both sides of carapace with 1 pair of frontal ridges and 3 pairs of oblique ridges (anterior, middle, and posterior); frontal ridges serrated, connecting to a transverse ridge; anterior oblique ridge beginning near pseudorostrum, running upward and not connecting to dorso-median portion of carapace, front part serrated; middle oblique ridge beginning near ventral margin of carapace, almost parallel with anterior oblique ridge, running upward and turning abruptly forward to merge with anterior oblique ridge; posterior oblique ridge running upward to 6/7 point of carapace, connecting with middle ridge; pseudorostral lobe and ocular lobe with 1 pair of small spines respectively, 2 small spines on median region of frontal lobe, lens of ocular lobe invisible; dorsal groove formed on posteromedian surface of carapace; antennal notch and antero-lateral angle prominent, antero-lateral and anterior half of lower margins serrated. Pereon (Fig. 2A, C) 0.7 times as long as carapace. Pleon (Fig. 2A) 0.8 times as long as carapace and pereon together.

Antenna 1 (Fig. 2D) peduncle 3-articulate; article 1 subequal in length to remaining articles combined, with several hair-like and 1 bent simple setae laterally, 7 slender simple setae on surface, numerous hair-like setae on surface and medial margin, 1 long plumose seta mediodistally; article 2 0.4 times as long as article 1, with several hair-like setae on both margins, 1 slender simple seta on surface, 2 simple, 1 long simple, and 2 plumose setae medially; article 3 1.7 times as long as article 2, with 2 long simple setae laterodistally, 2 slender simple setae on surface, 4 simple setae medially, 2 complex pedunculate setae mediodistally. Main flagellum 3-articulate; article 1 unarmed; article 2 with 1 aesthetasc distally; article 3 with 1 aesthetac, 3 short simple, 1 complex pedunculate, and 1 long annulate setae terminally. Accessory flagellum 3-articulate; article 1 with 1 short simple seta medially; article 2 with 1 complex pedunculate seta distally; 3 simple and 1 complex pedunculate setae terminally.

Left mandible (Fig. 2E) with row of 11 lifting setae; incisor with 2 teeth; lacinia mobilis with 3 teeth.

Right mandible (Fig. 2F) with row of 12 lifting setae; incisor with 2 teeth.

Maxilla 1 (Fig. 2G) outer endite with 1 plumose seta laterally, several hair-like setae medially, 7 stout simple, 3 stout microserrate, and several hair-like setae terminally; inner endite with several hair-like setae lateral and medial margins, 1 simple, 2 stout simple, 1 stout tricuspid, and 1 stout microserrate setae terminally; palp with 2 setae.

Maxilla 2 (Fig. 2H) broad endite with few hair-like setae laterally, several hair-like, 21 simple, and 2 microserrate setae medially, several hair-like, 1 stout pappose, 3 microserrate, 1 plumose, and 13 simple setae terminally; outer endite with 5 stout microserrate setae terminally; inner endite with 4 stout microserrate setae terminally.

Maxilliped 1 (Fig. 3A) basis with numerous hair-like and 3 short simple setae on lateral surface, 1 plumose seta mediodistally, medial lobe with 5 plumo-microserrate, 3 plumose, and 2 hook setae medially, 1 plumose, 2 stout simple, 1 simple, and 1 stout bicuspid setae terminally; ischium not present; merus with few hair-like setae laterally, several hair-like and 1 plumose setae on lateral surface, 2 plumose setae distally; carpus with few hair-like and 1 long plumose setae laterally, several hair-like and 7 plumose setae on lateral surface, 4 comb-like setae medially, 4 plumose setae subdistally; propodus with 5 plumose, 2 long plumose, 1 stout microserrate, and 1 stout serrate setae distally; dactylus with few hair-like setae laterally, 4 simple setae terminally.

Maxilliped 2 (Fig. 3B) basis subequal in length to remaining articles combined, with several hair-like setae on lateral and medial margins, 3 short simple setae on medial surface, 4 long plumose setae distally; ischium short, unarmed; merus with 2 plumose setae distally; carpus with several hair-like and 5 plumose setae medially, 3 plumose setae distally; propodus with 2 plumose and 2 plumo-serrate setae medially, 2 plumose setae on medial surface, 1 plumose and 1 long plumose setae distally; dactylus with 4 simple, 2 stout simple, and 1 microserrate setae terminally.

Maxilliped 3 (Fig. 3C) basis 1.4 times as long as remaining articles combined, with 12 slender simple setae on lateral surface, numerous hair-like setae on surface and both margins, 1 short simple, 1 plumose, and 4 long plumose setae laterodistally, several spines, 1 stout spiniform and 7 plumose setae medially; ischium with 4 slender simple setae on lateral surface, 1 stout spiniform, several hair-like, and 1 plumose setae medially; merus 1.3 times as long as ischium, with 1 long plumose seta laterally, 1 stout spiniform, several hair-like and 2 plumose setae medially; carpus 1.3 times as long as merus, with 1 plumose seta laterally, 2 long simple and 3 plumose setae medially; propodus 0.8 times as long as carpus, with 1 plumose seta laterally, 4 plumose setae medially; dactylus 0.7 times as long as propodus, with 2 long simple setae laterodistally, 2 microserrate setae mediodistally, and 1 simple, 1 long stout simple, and 1 long microserrate seta terminally; exopod shorter than basis length.

Pereopod 1 (Fig. 3D) basis 0.4 times as long as remaining articles combined, with 9 slender simple setae on surface, 9 plumose setae on lateral surface and margin, 1 complex pedunculate and numerous hair-like setae on lateral surface, several spines, 4 short simple, 13 simple, and 10 plumose setae medially, 2 short simple and 4 long plumose setae distally; ischium 0.1 times as long as basis, with few hair-like and 1 short simple setae medially; merus 2.6 times as long as ischium, with several hair-like, 1 short simple, and 2 plumose setae laterally, 3 slender simple and 1 plumose setae on surface, 1 short simple and 2 simple setae medially; carpus 2.3 times as long as merus, with 1 short simple and 2 simple setae laterally, 12 slender simple setae on lateral surface, numerous hair-like and 2 simple setae medially; propodus 1.2 times as long as carpus, with 7 simple setae laterally, 12 slender simple setae on surface, numerous hair-like, 4 simple, and 4 long simple setae medially; dactylus 0.4 times as long as propodus, with 2 short simple and 4 simple setae laterally, few hair-like, 1 short simple, and 7 simple setae medially, 2 simple and 2 stout simple setae terminally; exopod shorter than basis length, basal article with several hair-like and 1 short simple setae laterally.

Pereopod 2 (Fig. 4A) basis subequal in length to remaining articles combined, with 12 slender simple and 1 complex pedunculate setae on lateral surface, 9 short simple setae on lateral surface and margin, 4 spines, 17 simple, 4 plumose, and 8 long plumose setae medially; ischium 0.1 times as long as basis, with few hair-like setae laterally, few hair-like and 1 short simple setae medially; merus 2.0 times as long as ischium, with few hair-like, 1 slender simple, 2 short simple, and 1 plumose setae laterally, few hair-like, 1 short simple, and 1 simple setae medially, 1 plumose seta distally; carpus 1.3 times as long as merus, with few hair-like, 1 slender simple, 1 short simple and 1 simple setae laterally, 3 short simple and 2 simple setae medially; propodus 0.6 times as long as carpus, with few hair-like setae laterally, 1 complex pedunculate, 2 short simple and 1 long simple setae distally; dactylus subequal in length to propodus, with few hair-like, 1 short simple, and 1 simple setae laterally, few hair-like, 3 simple, and 1 annulate setae medially, 1 short simple and 4 annulate setae terminally; exopod longer than basis length, basal article with several hair-like and 6 short simple setae laterally.

Pereopod 3 (Fig. 4B) basis 1.2 times as long as remaining articles combined, with 5 slender simple setae on lateral surface, 6 simple and 1 complex pedunculate setae laterally, 1 simple, 2 plumose, and 3 long plumose setae on medial surface, 1 simple and 1 complex pedunculate setae medially; ischium 0.1 times as long as basis, with 1 simple seta laterally, 1 simple seta medially, 1 simple and 2 annulate setae mediodistally; merus 2.9 times as long as ischium, with 2 slender simple setae on lateral surface, 3 simple setae laterally, 3 simple setae medially, 2 simple setae on surface; carpus 0.5 times as long as merus, with 1 simple, 1 annulate, and 1 stout annulate setae distally; propodus 0.9 times as long as carpus, with 1 spine laterally, 1 complex pedunculate seta medially, 1 stout annulate seta distally; dactylus 0.5 times as long as propodus, with 1 simple seta medially, 1 simple and 1 stout simple setae terminally.

Pereopod 4 (Fig. 4C) basis 0.8 times as long as remaining articles combined, with 2 slender simple setae on surface, 2 simple setae laterally, 1 simple and 2 annulate setae laterodistally, 3 simple and 2 complex pedunculate setae medially; ischium 0.2 times as long as basis, with 1 simple and 1 annulate setae laterally; merus 3.1 times as long as ischium, with 1 slender simple seta on surface, 2 simple and 1 annulate setae laterally; carpus 0.3 times as long as merus, with 1 annulate seta laterally, 3 annulate setae medially, 1 simple seta distally; propodus 0.9 times as long as carpus, with 1 annulate seta on lateral surface, 1 complex pedunculate seta mediodistally; dactylus 0.6 times as long as propodus, with 1 simple seta medially, 1 simple and 1 stout simple setae terminally.

Pereopod 5 (Fig. 4D) basis 0.7 times as long as remaining articles combined, with 1 slender simple, 1 simple, 1 annulate, and 1 complex pedunculate setae on lateral surface, 2 simple and 1 annulate setae laterally, 1 simple seta mediodistally, 1 annulate seta distally; ischium 0.2 times as long as basis, with 1 simple seta medially, 2 annulate setae distally; merus 3.7 times as long as ischium, with 2 annulate and 1 simple setae laterally, 1 simple seta medially; carpus 0.6 times as long as merus, with 2 annulate setae laterally, 3 annulate setae on lateral surface, 1 simple seta medially; propodus 0.6 times as long as carpus, with 1 complex pedunculate and 1 annulate setae distally; dactylus 0.8 times as long as propodus, with 1 simple seta medially, 1 simple and 1 stout simple setae terminally.

Telson (Fig. 4E) 0.8 times as long as pleonite 6, with 7 slender simple setae on surface, 1 pair of short simple and 1 short stout simple seta centrally, 1 pair of stout simple and 3 pairs of short simple setae on post-anal section, 2 stout simple setae terminally.

Uropod (Fig. 4E) peduncle 2.8 times as long as telson, 2.4 times as long as pleonite 6, with 10–13 slender, simple setae on surface, 5–6 short simple setae laterally, 7 simple setae bearing single subterminal setule and numerous hair-like setae medially. Uropod endopod 3-articulate, 0.4 times as long as peduncle; article 1 subequal in length to remaining articles combined, with 2 simple setae bearing single subterminal setule and several hair-like setae medially, 1 slender simple seta on surface, 1 short simple and 1 complex pedunculate setae laterally; article 2 with 1 simple seta bearing single subterminal setule and several hair-like setae medially, 1 short simple seta laterally; article 3 with 1 simple and several hair-like setae medially, 1 short simple seta laterally, 1 long stout simple seta terminally. Uropod exopod 2-articulate, 0.9 times as long as endopod; article 1 short, with 1–2 short, simple setae laterally; article 2 with 1 simple seta mediodistally, 3–4 short simple setae laterally, 1 microserrate and 1 long stout simple seta terminally.

**Paratype, adult male, cat no. NIBRIV0000911250.** Body (Fig. 6A) length 4.45 mm. Body surface (Fig. 5) covered with numerous slender, simple setae. Carapace (Fig. 6A, C) 0.3 times as long as body, 1.5 times as long as width, 1.9 times as long as depth, ridge pattern similar to females, ridges not serrated; pseudorostral lobe and ocular lobe without spines; ocular lobe with 3 distinct lenses; antennal notch and antero-lateral angle prominent, antero-lateral margin serrated. Pereon (Fig. 6A, C) 0.6 times as long as carapace. Pleon (Fig. 6A) 0.8 times as long as carapace and pereon together; pleonite 5 with 1 spiniform seta on ventral margin.

Antenna 1 (Fig. 6D) peduncle 3-articulate; article 1 slightly longer than remaining articles combined, with numerous hair-like, 1 slender simple, and 4 short simple setae on surface, 1 bent simple seta on medial surface, numerous hair-like and 1 simple seta medially, 1 spiniform and 1 plumose seta mediodistally; article 2 0.4 times as long as article 1, with 1 short simple and 3 plumose setae on surface, 1 long simple seta medially; article 3 1.1 times as long as article 2, with 1 simple seta laterally, 3 short simple, 3 simple, and 3 complex pedunculate setae medially, brush of setae subterminally. Main flagellum 5-articulate; article 1 unarmed; article 2 with 2 simple setae distally; article 3 with 1 simple seta distally; article 4 with 1 aesthetasc distally; article 5 with 1 aesthetasc, 2 short simple and 1 complex pedunculate setae terminally. Accessory flagellum 4-articulate; article 1 unarmed; article 2 with 1 short simple seta medially, 3 simple setae distally, article 3 unarmed; article 4 with 2 short simple and 1 long simple seta terminally.

Antenna 2 (Fig. 6E) elongated, extending beyond end of telson; peduncle 5-articulated, article 1 and article 2 with 1 plumose seta each, article 4 with 1 short simple seta, article 5 with ranks of simple setae; each article of flagellum with several simple setae.

Maxilliped 3 (Fig. 6F) basis 1.9 times as long as remaining articles combined, with numerous hair-like setae laterally, 1 plumose and 4 long plumose setae laterodistally, 14 slender simple and 6 short simple setae on lateral surface, 9 spines, 1 stout spiniform, and 8 plumose setae medially; ischium with 1 slender simple seta on lateral surface, 1 plumose seta laterally, 1 stout spiniform, few hair-like, and 1 plumose setae medially; merus 1.4 times as long as ischium, with few hair-like and 1 long plumose setae laterally, 1 stout spiniform, few hair-like, and 3 plumose setae medially; carpus 0.9 times as long as merus, with 1 plumose seta laterodistally, few hair-like, 1 simple, and 3 plumose setae medially; propodus 1.4 times as long as carpus, with 1 plumose seta laterodistally, few hair-like and 4 plumose setae medially; dactylus 0.6 times as long as propodus, with 1 microserrate and 2 simple setae laterodistally, 1 microserrate, 2 simple, and 2 long microserrate setae terminally; exopod shorter than basis length, basal article with 2 short simple setae laterally, 8 slender simple setae on lateral surface, 4 plumose setae medially.

Pereopod 1 (Fig. 6G) basis 0.6 times as long as remaining articles combined, with 17 slender simple setae on lateral surface, 14 plumose setae on lateral surface and margin, 3 long plumose setae laterodistally, 12 spines, 4 simple and 15 plumose medially, 3 plumose and 1 long plumose setae distally; ischium 0.1 times as long as basis, with 1 short simple seta medially; merus 1.8 times as long as ischium, with 8 slender simple setae on lateral surface, 6 slender simple setae on medial surface, 1 simple and 1 plumose seta laterally, 2 short simple setae medially; carpus 2.5 times as long as merus, with 11 slender simple setae on lateral surface, 8 slender simple setae on medial surface, 1 short simple seta laterally, 1 short simple and 1 simple setae medially; propodus 1.2 times as long as carpus, with 7 slender simple setae on lateral surface, 8 slender simple setae on medial surface, 6 simple setae laterally, 6 simple and 1 long simple setae medially; dactylus 0.4 times as long as propodus, with 2 short simple and 1 simple setae laterally, 6 simple setae medially, 1 simple and 3 stout simple setae terminally; exopod shorter than basis length, basal article with 15 slender simple setae on lateral surface, 1 short simple and 1 plumose setae laterodistally, 4 plumose setae medially.

Pereopod 2 (Fig. 7A) basis 1.4 times as long as remaining articles combined, with 6 plumose setae laterally, 15 slender simple, 1 simple, and 5 plumose setae on surface, 2 simple and 5 plumose setae medially; distal corner very inflated, reaching to the middle of carpus, with 10 plumose setae; ischium short, unarmed; merus 2.1 times as long as ischium, with 1 short simple and 1 plumose seta laterodistally, 1 slender simple seta on lateral surface, 1 short simple and 1 plumose setae mediodistally; carpus 1.4 times as long as merus, with 2 short simple setae laterally, 1 slender simple seta on lateral surface, 1 short simple and 3 simple setae medially; propodus 0.6 times as long as carpus, with 1 slender simple seta on lateral surface, 1 complex pedunculate, 2 short simple, and 1 long simple setae distally; dactylus 1.3 times as long as propodus, with 1 short simple and 1 simple seta laterally, 2 short simple, 2 simple, and 1 long simple setae medially, 1 short simple, 2 simple, and 1 long simple setae terminally; exopod longer than basis length, basal article with 2 short simple setae laterally, 9 slender simple setae on lateral surface, 2 simple setae distally, 2 plumose setae medially.

Pereopod 3 (Fig. 7B) basis 1.3 times as long as remaining articles combined, with 7 slender simple, 1 complex pedunculate, 1 short simple, 2 short plumose, and 3 plumose setae on lateral surface, 4 plumose setae on medial surface, 1 simple and 3 plumose setae medially; distal corner very inflated, reaching to the one-third of merus, with 3 plumose setae; ischium 0.1 times as long as basis, with 2 simple and 1 annulate setae; merus 3.9 times as long as ischium, with 3 simple setae laterally, 1 simple seta on medial surface, 1 short simple seta medially; carpus 0.5 times as long as merus, with 2 annulate setae laterally, 1 simple and 2 annulate setae on lateral surface, 1 simple seta on medial surface, 1 simple seta mediodistally; propodus 0.7 times as long as carpus, with 1 annulate and 1 complex pedunculate setae distally; dactylus 0.7 times as long as propodus, with 1 simple and 1 stout simple seta terminally; exopod slightly shorter than basis length, basal article with 1 simple seta laterally, 1 plumose seta laterodistally, 10 slender simple setae on lateral surface, 1 short simple and 1 plumose seta medially.

Pereopod 4 (Fig. 7C) basis 1.3 times as long as remaining articles combined, with 18 slender simple, 7 plumose, 4 complex pedunculate, and 1 short simple seta on surface, several hair-like setae medially; distal corner very inflated, reaching to the one-third of merus, with 1 short simple, 2 simple, and 5 plumose setae; ischium 0.1 times as long as basis, with 1 simple and 1 annulate seta medially; merus 3.5 times as long as ischium, with 2 slender simple setae on lateral surface, 4 short simple setae laterally, 3 simple setae medially; carpus 0.4 times as long as merus, with 1 slender simple seta on lateral surface, 2 annulate setae laterally, 1 short simple and 2 simple setae medially, 2 annulate setae distally; propodus subequal in length to carpus, with 1 annulate seta laterodistally, 1 complex pedunculate seta mediodistally; dactylus 0.5 times as long as propodus, with 1 short simple seta medially, 1 simple and 1 stout simple setae terminally; exopod longer than basis length, basal article with 2 short simple and 4 simple setae laterally.

Pereopod 5 (Fig. 7D) basis 0.6 times as long as remaining articles combined, with few hair-like setae laterally, 1 complex pedunculate and 3 annulate setae laterodistally, 6 slender simple and 1 plumose setae on lateral surface, 1 annulate and 1 plumose seta medially; ischium 0.2 times as long as basis, with 1 short simple and 3 annulate setae laterally; merus 2.9 times as long as ischium, with 1 simple and 1 annulate seta laterally, 1 annulate seta on lateral surface, 1 short simple seta medially; carpus 0.7 times as long as merus, with 1 simple and 2 annulate setae laterally, 1 simple and 3 annulate setae medially; propodus 0.6 times as long as carpus, with 1 complex pedunculate and 1 annulate seta distally; dactylus 0.7 times as long as propodus, with 1 short simple seta medially, 1 simple and 1 stout simple setae terminally.

Pleopod 1 (Fig. 7E) basis with 3 microserrate and 3 plumose setae medially, 5 simple setae on lateral surface and margin; outer ramus with 4 long plumose setae terminally; inner ramus with 3 plumose setae medially, 4 long plumose setae terminally.

Pleopod 2 (Fig. 7F) basis with 3 microserrate setae medially; outer ramus 2-articulated, article 2 with 1 stout simple seta laterally, 3 long plumose setae terminally; inner ramus unarticulated, with 4 long plumose setae terminally.

Telson (Fig. 7G) 1.3 times as long as pleonite 6; pre-anal section with 6 slender simple and 4 short simple setae on surface, 24 complex pedunculate setae on U-shaped dorsal ridge; post-anal section with 2 pairs of stout simple setae bearing single subterminal setule on both sides, terminal margin with 3 stout simple setae, central seta slightly longer than outer pair of setae.

Uropod (Fig. 7G) peduncle 2.0 times as long as telson, 2.6 times as long as pleonite 6, with 14–27 slender simple setae on lateral surface and margin, 5 short simple setae laterally, numerous hair-like, 2 short simple setae, and 17–19 stout microserrate setae bearing single subterminal setule medially; Uropod endopod 3-articulate, 0.4 times as long as peduncle; article 1 1.2 times as long as remaining articles combined, with 2 complex pedunculate and 1 shot simple setae laterally, numerous hair-like setae and 6 stout microserrate setae bearing single subterminal setule medially; article 2 with 1 short simple seta laterally, several hair-like setae and 2 stout microserrate setae bearing single subterminal setule medially; article 3 with several hair-like and 1 stout microserrate seta bearing single subterminal setule medially; 1 short simple, 1 simple, and 1 long simple seta terminally. Uropod exopod 2-articulate, 0.9 times as long as endopod; article 1 short, with 1 short simple seta laterally, 2 simple setae laterally, 3 simple and 1 long simple seta terminally.

#### Remarks.

The most similar species is *Dimorphostylisasiatica* Zimmer, 1921, which has transverse, a pair of frontal and three pairs of oblique ridges on the carapace. However, the new species is obviously distinguished from *D.asiatica* by a combination of the following features (*D.asiatica* condition in parentheses): 1) body surface covered with numerous slender simple setae (vs. without slender simple setae); 2) carapace, posterior oblique ridge running upward to 6/7 point of carapace, connecting with the middle ridge (vs. posterior oblique ridge running upward and turning abruptly forward to merge with dorsal submedian carina); and 3) telson of female with 1 pair of short simple setae centrally, 1 pair of stout simple setae on post-anal section (vs. without a stout simple seta).

#### Etymology.

The new species name, *pilocorpus*, is from the combination of the Latin words *pilósus*, meaning ‘hairy or shaggy’ and *corpus*, meaning ‘body’, alluding to the body surface covered with numerous slender simple setae.

#### Habitat.

The new species was collected in Dokdo and Ulleung Islands (East Sea), Korea, which has a sandy substrate.

#### Depth.

5–20 m.

#### Distribution.

Korea (Dokdo and Ulleung Islands).

### ﻿Key to the species of *Dimorphostylis* from Korean waters (female of *D.hirsuta* unknown)

**Table:** 

1	Carapace, frontal lobe with transverse ridge	**2**
–	Carapace, frontal lobe without transverse ridge	**6**
2	Telson of male, post-anal section with more than a pair of short stout setae on both sides	**3**
–	Telson of male, post-anal section without short stout setae on both sides	**5**
3	Body surface covered with numerous slender simple setae	***D.pilocorpus* sp. nov.**
–	Body not covered with slender simple seta	**4**
4	Telson of female almost 0.7 times as long as pleonite 6, post-anal section with 3 pairs of lateral bristles, telson of male with 3 terminal setae of which middle seta longest	***D.asiatica***
–	Telson of female about 0.8 times as long as pleonite 6, post-anal section with 2–3 pairs of hairy setae near middle portion, 1–3 pairs of bristles, telson of male with 3 terminal setae of which middle seta very small	***D.namhaedoensis***
5	Uropod peduncle of male less than 2.5 times as long as telson, with 14–18 setae on medial margin	***D.hirsuta***
–	Uropod peduncle of male about 2.7 times as long as telson, with 13 setae on medial margin	***D.valida***
6	Carapace without frontal ridge	***D.manazuruensis***
–	Carapace with 1 pair of frontal ridges	**7**
7	Carapace, middle oblique ridge short, not connecting with anterior oblique ridge	***D.breviplicata***
–	Carapace, middle oblique ridge long, running upward and connecting with anterior oblique ridge	**8**
8	Uropod peduncle less than 2.0 times as long as telson	***D.longicauda***
–	Uropod peduncle more than 2.1 times as long as telson	**9**
9	Carapace, anterior oblique ridge W-shaped and not parallel with middle oblique ridge; propodus of pereopod 1 with 1 long and 9 short simple setae in female (with 1 long and 8 short simple setae in male)	***D.acroplicata***
–	Carapace, anterior oblique ridge curved and almost parallel with middle oblique ridge; propodus of pereopod 1 with 8 long and 12 short simple setae in female (with 8 long and 10 short simple setae in male)	***D.echinata***

## Supplementary Material

XML Treatment for Dimorphostylis

XML Treatment for Dimorphostylis pilocorpus

## ﻿Additional information

### Author ORCIDs

Sung-Hyun Kim https://orcid.org/0000-0002-0648-5227

Taekjun Lee https://orcid.org/0000-0003-4407-7862

### Data availability

All of the data that support the findings of this study are available in the main text.
